# Peptide therapy in sepsis and inflammation: a novel strategy to suppress inflammation

**DOI:** 10.1186/ar3595

**Published:** 2012-02-09

**Authors:** Hidechika Okada, Alan Okada

**Affiliations:** 1 Research Institute for Protein Science, Nakayama-cho, Mizuho-ku, Nagoya, Japan

## Antisense homology box (AHB)

In 1984, Blalock proposed the possible role of antisense peptides for molecular interaction among proteins (BBRC, *121*: 203, 1984).

We speculated that interactions between sense- and antisense-peptides should play a role in formation of the tertiary structure of proteins. We developed a novel computer program named ANTIS to find antisense peptide sequences between proteins to be compared (Nature Med. 1:894,1995). ANTIS revealed the presence of an appreciable number of sense and antisense peptide pairs within any protein molecule and those portions were designated as antisense homology boxes (AHB).

## Complementary peptide

Each peptide should have specific structure determined by its amino acid sequence which may react with its antisense peptide. To generate candidates of complementary peptide (C-pep) reactive to a target amino acid sequence based upon the sense-antisense amino acid relationship. We invented an evolutionary computer program (MIMETIC) that generates C-pep sequences that have a potential to interact with a target peptide (Microbiol. Imm.46:211, 2002).

## C5a inhibitory peptides

C5a anaphylatoxin is considered to be an effective target for treatment of hyperinflammation since C5a stimulates generation of tumor necrosis factor alpha (TNFα and other inflammatory cytokines. Amino acids 37 to 53 of C5a (RAARISLGPRCIKAFTE) is an antisense peptide to AHBpeptides of the C5a receptor (C5aR), and this has been designated PL37. This region of C5a is presumed to be a potential site for C5aR stimulation. Using the computer program MIMETIC, we generated 19 C-peps to PL37. One of the 7 inhibitory C-peps to PL37 which interfered with C5a function was termed PepA(ASGAPAPGPAGP- LRPMF). To improve stability, we modified PepA by acetylation of its *N*-terminal alanine generating acetylated PepA (AcPepA).

AcPepA rescued Cynomolgus monkeys at lethal shock induced by bacterial LPS (4 mg/kg).The excellent therapeutic effect of AcPepA is due to restriction of high mobility group box 1 (HMGB1) surge induced by the effect of C5a on C5L2, which is the second C5a receptor, since the released HMGB1 has the capacity to stimulate TLR4 as an endogeneous ligand resulting in further activation of inflammatory cells to release inflammatory cytokines forming positive feedback circuit of inflammation.

